# Heart Rate Variability Impairment Is Associated with Right Ventricular Overload and Early Mortality Risk in Patients with Acute Pulmonary Embolism

**DOI:** 10.3390/jcm12030753

**Published:** 2023-01-17

**Authors:** Monika Lisicka, Marta Skowrońska, Bartosz Karolak, Jan Wójcik, Piotr Pruszczyk, Piotr Bienias

**Affiliations:** 1 Department of Internal Medicine and Cardiology, Medical University of Warsaw, 02-005 Warsaw, Poland; 2 Students’ Scientific Association Zator, Department of Internal Medicine and Cardiology, Medical University of Warsaw, 02-005 Warsaw, Poland

**Keywords:** acute pulmonary embolism, electrocardiography, Holter monitoring, heart rate variability, right ventricle overload

## Abstract

The association between heart rate variability (HRV) and mortality risk of acute pulmonary embolism (APE), as well as its association with right ventricular (RV) overload is not well established. We performed an observational study on consecutive patients with confirmed APE. In the first 48 h after admission, 24 h Holter monitoring with assessment of time-domain HRV, echocardiography and NT-proBNP (N-terminal pro-B-type natriuretic peptide) measurement were performed in all participants. We pre-examined 166 patients: 32 (20%) with low risk of early mortality, 65 (40%) with intermediate–low, 65 (40%) with intermediate–high, and 4 (0.02%) in the high risk category. The last group was excluded from further analysis due to sample size, and finally, 162 patients aged 56.3 ± 18.5 years were examined. We observed significant correlations between HRV parameters and echocardiographic signs of RV overload. SDNN (standard deviation of intervals of all normal beats) correlated with echocardiography-derived RVSP (right ventricular systolic pressure; r = −0.31, *p* = 0.001), TAPSE (tricuspid annulus plane systolic excursion; r = 0.21, *p* = 0.033), IVC (inferior vena cava diameter; r = −0.27, *p* = 0.002) and also with NT-proBNP concentration (r = −0.30, *p* = 0.004). HRV indices were also associated with APE risk stratification, especially in the low-risk category (r = 0.30, *p* = 0.004 for SDNN). Univariate and multivariate analyses confirmed that SDNN values were associated with signs of RV overload. In conclusion, we observed a significant association between time-domain HRV parameters and echocardiographic and biochemical signs of RV overload. Impaired HRV parameters were also associated with worse a clinical risk status of APE.

## 1. Introduction

Venous thromboembolism, clinically presenting as deep vein thrombosis or acute pulmonary embolism (APE), is globally the third most frequent acute cardiovascular syndrome. Even though the annual fatality rates of the disease across Europe and Northern America have decreased over a 15 year period, APE is still associated with adverse clinical outcomes, including deterioration and death [1]. Numerous electrocardiographic abnormalities are observed during APE, especially in the 12-lead ECG. However, the evidence on the role of 24 h Holter or other long-term electrocardiography monitoring in controlling the clinical status or evaluating the prognosis of APE is scarce. This also applies to the heart rate variability (HRV) obtained during Holter monitoring, which is a reflection of cardiac autonomic nervous system (cANS) activity, both sympathetic and parasympathetic. Several studies revealed that cANS imbalances are associated with a worse clinical prognosis in patients with impaired left ventricular function, especially as a consequence of myocardial infarction or heart failure [2]. It is postulated that the sympathetic–parasympathetic imbalance might be associated with various adverse events, including severe and life-threatening ventricular arrhythmias [2,3]. The right side of the heart, especially the right atrium, is an area with even more autonomous innervation than the left side. Thus, HRV analysis is presumed to be especially effective in monitoring autonomic function in patients with conditions affecting mainly the right side of the heart, such as APE [3]. A potential model, linking HRV parameters with echocardiographic features of right ventricular (RV) overload may lead to improved prognosis assessment, and consequently, improvement of clinical practice in these patients. However, the clinical utility of HRV has not yet been established in large cohorts of APE patients. 

We make the assumption that time-domain HRV analysis, which can be easily performed by standard Holter monitoring [4], might be a promising tool for risk stratification of patients with APE. Therefore, the aim of our study was to evaluate the relationship between Holter-derived HRV indices and specific echocardiographic and laboratory parameters, well established in the assessment of the prognosis of APE, along with their analysis in the context of APE risk stratification according to the European Society of Cardiology (ESC) [1].

## 2. Materials and Methods

We performed a prospective, single-center, cross-sectional, observational study, running for 10 years between 2009 and 2019. At baseline, consecutive adult patients with APE confirmed by imaging (mostly computed tomography pulmonary angiogram) were included. Detailed evaluation of clinical status, past medical history and laboratory tests were performed at admission, while echocardiography and 24 h Holter monitoring were conducted within 48 h of admission at the latest (most often <24 h). Then, the prognosis of APE was assessed according to the ESC guidelines. All participants were assessed according to current ESC guidelines but, in terms of the present data analysis, retrospectively assigned to risk categories stated in the 2019 guidelines, i.e., low, intermediate–low, intermediate–high and high risk. This classification provides information about the APE severity and the risk of early (in-hospital or 30-day) mortality. Patients with clinical signs of hemodynamic instability were classified in the high risk category. Patients without hemodynamic instability, but with presence of both RV dysfunction (on echocardiography or computed tomography angiography) and elevation of laboratory biomarkers (mainly cardiac troponin, but N-terminal pro-B-type natriuretic peptide (NT-proBNP) also provides prognostic information) were referred to the intermediate–high risk category. The presence of only one of the aforementioned signs of RV disfunction (in cardiac imaging or biochemical) enabled the identification of the intermediate–low risk category, while the absence of both of them to the low risk category, provided that the simplified pulmonary embolism severity index score was 0 points [1].

Transthoracic echocardiography was performed using a Philips iE33 ultrasound system (Philips Medical System, Andover, MA, USA) with a 2.5–3.5 MHz transducer. Patients were examined in the left lateral position. According to current American and European recommendations, all standard dimensions, valve morphology and function, left ventricular ejection fraction and right heart parameters were evaluated [5]. The emphasis was put on the RV assessment, including following parameters: TRPG (tricuspid regurgitation peak gradient), RVSP (right ventricular systolic pressure), AcT (right ventricular outflow Doppler acceleration time), IVC (inferior vena cava diameter) and TAPSE (tricuspid annulus plane systolic excursion) measurement. Considering the extensive experience of our experts in the field of diagnostic imaging, verified by the certificate of the Section of Echocardiography of the Polish Cardiac Society, which is a part of the ESC, as well as the high repeatability (inter/intra-observer), which was confirmed in one of the studies on patients with APE carried out in our center, echocardiographic examination was performed at a time by one experienced physician [6].

Ambulatory Holter monitoring was conducted using three-channel digitized recordings (Lifecard CF, Spacelabs Healthcare, Snoqualmie, WA, USA). Routine evaluations of heart rates and arrhythmias were performed (Sentinel Impresario, Spacelabs Healthcare, USA). Careful manual review and filtering algorithms were used to eliminate inappropriate electrocardiographic strips, aimed to eliminate fragments illegible for further analysis. Time-domain HRV parameters were obtained from 24 h Holter monitoring, including SDNN (standard deviation of intervals of all normal beats), SDNN-I (the average of the standard deviations of N-N intervals for each 5-min), SDANN (standard deviation of five-minute mean N-N interval), RMSSD (root mean square standard deviation), pNN50 (percentage of intervals that are more than 50 ms different from previous interval) and triangular index (integral of the density of the NN interval histogram divided by its height) [4]. These parameters were chosen as sufficient for time-domain HRV analysis, considering that many parameters closely correlate with others. SDNN and HRV-Index estimate overall HRV, SDANN and SDNN-I estimate long-term components of HRV, RMSSD and pNN50 estimate short-term components of HRV [4]. For each HRV parameter, the results of the entire 24 h evaluation were taken for further analysis, without the division into day and night periods. Each Holter recording was analyzed by a qualified and certified cardiologist without blinding.

Echocardiographic and Holter examinations were performed by different members of the research staff who knew the full protocol but did not know the detailed results obtained, which were later transferred to the database. Taking in the relatively modest data on long-term electrocardiographic monitoring and Holter-derived cANS assessment in APE patients, we decided to conduct an exploratory study to provide new information on this topic without a pre-existing hypothesis.

## 3. Statistical Analysis and Data Presentation

The data collection was not blinded. Continuous variables were compared with Student’s *t* test or the Wilcoxon test, according to data distribution. Categorical variables were compared using Fisher’s exact test. All tests were double-sided. Variables with a normal distribution are presented as mean followed by standard deviation. Deletions of outliers’ data were not performed. Variables not showing normal distribution, as well as those with skewed distribution, were presented as median with range and also with interquartile range values. Pearson’s correlation coefficient was used to assess the significance of the connections between parameters considered to show normal distribution. Abridged logistic regression analysis was performed to explore the influence of confounding factors on the values of SDNN, the most important HRV parameter. Both univariate and multivariate analyses were carried out with the examination of multiple models. Statistical significance was determined at *p* < 0.05. Results of univariate and multivariate analyses are presented as odds ratios (OR) with 95% confidence intervals (95% CI). All statistical analyses were performed using Statistica 14.0 (StatSoft Inc., Tulsa, OK, USA). 

## 4. Results

The study cohort consisted of 166 consecutive patients with confirmed APE admitted to our specialist center, almost all of them to our cardiac care unit. As described above, all participants were assigned to the APE risk classification groups according to ESC guidelines. The majority of the patients was allocated to the intermediate risk category (130 persons, 80%), of which exactly half belonged to the intermediate–low risk APE category (65 persons) and the other half to the intermediate–high risk APE. Low risk APE consisted of 32 (20%) patients, while only four (0.02%) participants had the high risk disease. Patients with the high clinical risk category were excluded from further statistical analysis due to small sample size, which cannot give conclusive results in the context of the whole study population. Then, to reach the needs of statistical equations and provide results, more plausible and representative for our study population, we merged the intermediate–low and intermediate–high risk groups into one group of patients with the intermediate risk APE. All patients received standard anticoagulation therapy and other medications needed for their specific comorbidities.

The general characteristics at baseline are shown in Table 1 and did not differ significantly between groups of patients with low and intermediate risk. Results were not different for males and females either—detailed results are not shown. As expected, due to right RV overload, the NT-proBNP level was significantly higher in intermediate risk APE. For the same reason, echocardiographic parameters related to impaired RV function were worse in this group of patients in comparison with the low risk APE population. During a follow-up of 48 h, none of the patients finally enrolled in the study died, required thrombolysis, or were transferred to an intensive/critical care unit.

The results of 24 h Holter monitoring in APE patients are also displayed in Table 1. For all APE patients, the median Holter recording time was 23 h and 22 min and was similar in the low and intermediate risk category groups (23:16 vs. 23:37, *p* = 0.82), while the shortest recording was 21 h and 12 min. However, the results of our HRV analysis are not obvious and easy to explain. While the values of SDNN, pNN50 and RMSSD were significantly lower in the intermediate than in the low risk APE group, no remarkable difference was found in the values of other parameters. 

**Figure 1:**
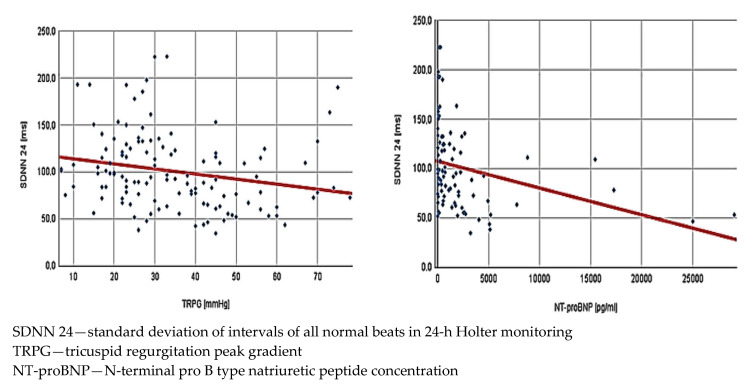
Correlations in all low and intermediate risk patients with acute pulmonary embolism (n = 162) between standard deviation of intervals of all normal beats in 24 h Holter monitoring and tricuspid regurgitation peak gradient (r = 0.22, *p* = 0.016)—left side of the figure—and N-terminal pro-B type natriuretic peptide concentration (r = −0.30, *p* = 0.004)—right side of the figure.

The next step was analysis of the potential relations between HRV and parameters obtained in echocardiography and biochemical tests. It was made collectively for the entire study population. Interestingly, while the mean HR was not associated with any of the echocardiographic measurements, its correlation with NT-proBNP levels reached statistical significance. We also found a lot of significant correlations between HRV and echocardiographic parameters describing RV function (Figure 1). Notably, most HRV parameters showed an inverse correlation with NT-proBNP concentration—detailed results are presented in Table 2. 

Moreover, we performed abridged univariate and multivariate logistic regression analysis to assess the association of the echocardiographic parameters and NT-proBNP concentration on the values of SDNN, the principal parameter in the HRV analysis. The results are presented in Table 3. In univariate analysis TRPG, RVSP, TAPSE, IVC, AcT and NT-proBNP were examined and all of them showed a significant association with SDNN values. However, in the multivariate analysis only IVC diameter and NT-proBNP level remained statistically relevant. 

## 5. Discussion

In our study we put impact on analyzing the connection between time-domain HRV and APE in the context of its severity and prognosis. We revealed novel associations between 24 h Holter monitoring HRV parameters and clinical status, as well as the echocardiographic and biochemical signs of RV overload. Extensive analysis of HRV parameters and their correlation with indices of RV overload have given us promising results with potential clinical value. The strength of our research is also a large cohort of patients observed in the severe phase of APE.

Over the last decades, HRV parameters were increasingly considered as indicators of the cANS activity [4,7,8]. However, there is limited research depicting HRV as an important indicator of cANS dysfunction in patients with RV disorders. Among others, Bienias et al. and Witte et al. demonstrated that Holter-derived parameters depict significant cANS impairment in patients with pulmonary hypertension of various etiologies [9,10,11,12]. Several studies have demonstrated that patients with arterial or chronic thromboembolic pulmonary hypertension are characterized by significant impairment of heart rate turbulence, which was related to the disease severity [9,13]. Patients with impaired RV due to pulmonary arterial hypertension were also found to have decreased post-exercise HRV parameters in comparison with healthy individuals, which reflects the prolonged recovery of cANS control in this population [14]. HRV parameters were also shown to be impaired in other chronic RV overload conditions, e.g., in arrhythmogenic right ventricular cardiomyopathy and tetralogy of Fallot [3,15]. In some studies the main and the simplest cANS parameter, i.e., mean HR, reflecting an increased sympathetic and decreased parasympathetic tone, was also found to be a prognostic factor in venous thromboembolism occurrence [16,17]. 

Lower values of HRV parameters can be considered to be predictors of various cardiac arrhythmia occurrences, including life-threatening ventricular arrhythmias and atrial fibrillation [2,18]. According to the literature, the most common serious arrhythmia appearing in the first days of APE is atrial fibrillation, followed by other supraventricular tachycardias [19,20,21]. Surprisingly, in our study population, atrial fibrillation was extremely uncommon, while short supraventricular tachycardias were recorded in 29% of patients but without clinical deterioration. It remains unclear whether this is an effect of the sample size or the reason for the relatively high proportion of patients in relatively good clinical status after admission. 

Clinical observations indicate that the occurrence of serious ventricular arrhythmias immediately after APE is very rare with unclear recommendations for long-term management and prognosis [22]. Cardiac arrest being a result of APE is usually related to pulseless electrical activity [3]. In our study no significant ventricular arrhythmias were found either. Syncope (usually as one of the first sign of the diseases) may occur in about 10% of patients with APE, it is mainly related to cANS dysfunction, and may worsen the prognosis [23,24]. As such, they are another potential coefficient, the risk of which could be identified using HRV parameters. Yet, in our study population we did not identify any cases of syncope during hospitalization. 

It is important to remark that most of the clinical characteristics of our participants did not differ significantly between groups with low and intermediate risk of APE. It is especially compelling that one half of the intermediate risk group consisted of patients with intermediate–high risk of APE. It is clear that RV overload in our cohort observed in diagnostic imaging did not cause major changes in the parameters describing the general status at admission and did not remarkably increase the risk of shock in the study population. 

Even though, echocardiographic parameters describing the RV function remarkably differed between groups, indicating that patients with the intermediate risk APE had significantly higher overload of RV, in spite of similar general status parameters at admission. Importantly, echocardiographic parameters describing the left ventricular function did not differ between low and intermediate risk APE groups, which provides a conclusion that cANS dysfunction observed in our patients was triggered mainly by RV overload. Supporting this suspicion is the fact that we revealed significant correlations between HRV parameters and echocardiographic-derived TRPG, RVSP, IVC, TAPSE and AcT (Table 2). Additional correlations between HRV indices and NT-proBNP level are also consistent. Logistic regression analysis confirmed that the increase in SDNN values was associated with better echocardiography-derived indices of RV function and lower values of NT-proBNP (in contrast, worse echocardiographic parameters and increased NT-proBNP concentration were associated with lower SDNN values). It is a novel finding, and to our knowledge, has not yet been described in the literature regarding APE. 

Most of the HRV parameters, including SDNN and SDANN, are assumed to express mainly sympathetic nervous system activity, whereas RMSSD and pNN50 were found to reflect rather the parasympathetic composition of the cANS [2,4]. Considering that in our study the relationship between the tested HRV parameters and APE risk status reached statistical significance, there is a need for further research in this area. Our results might indicate the prominent role of relative parasympathetic dysfunction in the course of APE.

## 6. Limitations

Our study also has several limitations. First, our study cohort consisted of only few individuals with high risk APE (we excluded them from the statistical analysis). Second, we observed a relatively good clinical status of patients with intermediate–high risk APE. It might have been a reason for the inconclusive incidence of arrhythmia and undetected syncope in the severe phase of APE during our observation, as well as the unclear results of comparison between different HRV parameters in both analyzed risk groups. Third, the relationship between HRV and echocardiographic markers of RV overload reached statistical significance, but there is no proven causality between them, which makes their results hypothesis-generating. Moreover, it remains unclear whether more and stronger associations between HRV parameters and APE prognosis could be found in larger study cohorts, with more representative proportions of individual APE risk categories, especially with more patients at high risk. Fourth, the observation time was short, up to 24–48 h after admission, with no follow-up in our protocol. Therefore, it is not possible to predict adverse events in the long-term. Fifth, in our analysis, we skipped the assessment of cardiac troponins because during the study the methods of laboratory assessment in our center, and thus, normal values changed (standard high-sensitive troponins). Sixth, another possible limitation is the lack of frequency-domain (power spectral) HRV analysis. However, we are convinced that well-performed time-domain HRV is sufficient for the first line cANS function evaluation. In addition, frequency-domain analysis should be performed under controlled registration conditions, preferably in a laboratory, similar for all patients, which was not possible and planned in our study protocol. It is important to take into account that all the conditions, such as concomitant clinical diseases, medications taken or the time of admission of the patient to the ward, will have a significant impact on the results of such an analysis and will make the interpretation of the results difficult and error-prone. 

## 7. Conclusions

We revealed a significant association between time-domain HRV parameters and echocardiographic, as well as biochemical signs of RV overload. Moreover, some HRV parameters were associated with the clinical risk classification of APE, differing significantly between groups with intermediate and low risk of early mortality. As Holter monitoring with HRV analysis is an easy-to-obtain and cost-effective diagnostic method, our observations indicate the need for further evaluation to determine the clinical significance and standardization of HRV analysis in patients with acute pulmonary embolism.

## Figures and Tables

**Table 1:** The general characteristics, laboratory data, echocardiographic parameters and 24 h Holter and time-domain heart rate variability data in all enrolled patients with acute pulmonary embolism (APE), as well as in subgroups with low and intermediate early mortality risk.

	All APE Patients	Low Risk APE	Intermediate Risk APE	*p* Value
	(*n* = 162) ^a^	(*n* = 32)	(*n* = 130)	Low vs. Intermediate Risk APE
**General characteristics and laboratory data at admission**				
Age (years) ^b^	56.3 ± 18.5	54.6 ± 17.2	53.4 ± 18.0	0.39
	(18–86)	(20–82)	(18–86)	
Females (*n*, %)	89 (54%)	18 (56%)	68 (52%)	0.8
BMI (kg/m^2^)	29.1 ± 5.6	27.2 ± 5.5	31.1 ± 5.7	**0.04**
HR at admission (bpm)	88 ± 18	87 ± 22	91 ± 16	0.21
SBP at admission (mmHg)	128.1 ± 18.8	128.0 ± 16.5	130.8 ± 18.7	0.48
NT-proBNP (pg/mL) *	627	95	1443	**0.02**
	(52.5–2795.5, 160.0–2321.0)	(18.0–272.0, 52.5–201.0)	(59.0–29,071.0, 486.0–2795.5)	
**Echocardiography parameters**				
LVEF (%)	59.7 ± 6.9	62.3 ± 2.6	59.5 ± 7.6	0.09
LVDD (mm)	41.4 ± 6.6	43.6 ± 6.3	41.2 ± 7.6	0.28
RVD (mm)	38.2 ± 9.0	32.8 ± 6.8	38.6 ± 8.0	**0.02**
TRPG (mmHg)	34.4 ± 16.3	21.6 ± 6.7	37.6 ± 15.5	**0.005**
RVSP (mmHg)	41.5 ± 17.3	27.0 ± 6.7	46.6 ± 16.0	**0.005**
IVC (mm)	15.5 ± 4.6	13.6 ± 2.9	16.2 ± 4.7	**0.02**
AcT (ms)	83.5 ± 27.5	105.3 ± 22.2	75.8 ± 26.5	**0.005**
TAPSE (mm)	21.1 ± 4.9	23.9 ± 3.7	20.5 ± 4.9	0.06
**24 h Holter and time-domain heart rate variability data**				
Mean heart rate (bpm)	77 ± 14	75 ± 11	79 ± 13	0.26
Non-sustained SVT (*n*, %)	47	5	41	0.3
	−29.00%	−15.70%	−31.50%	
Non-sustained VT (*n*, %)	9	2	7	0.4
	−5.40%	−6.30%	−5.40%	
Paroxysmal atrial fibrillation (*n*., %)	2	0	2	-
	−0.01%	-	−0.01%	
SDNN (ms) *	92	113.6	87.9	0.05
	(39.6–222.9, 68.8–121.4)	(65.5–222.9, 93–154.2)	(39.6–193.1, 65–116)	
SDNN-I (ms) *	30.2	35.7	28	0.06
	(10.1–90.4, 21.2–39.3)	(15.9–90.4, 32.9–52.4)	(10.1–85.9, 20.7–37.3)	
SDANN (ms) *	85.1	100.1	80.6	0.1
	(29.0–190.4, 61.0–110.8)	(35.2–190.4, 82.6–126.9)	(29.0–182.9, 58–107.9)	
RMSSD (ms) *	26.8	32.8	26.8	**0.02**
	(12.4–96.2, 20.2–38.2)	(12.6–96.2, 22.9–54)	(12.4–94.2, 19.9–36.9)	
pNN50 (%) *	2.8	6.8	2.45	**0.03**
	(0.1–74.8, 1.0–16.0)	(0.1–74.8, 2.1–16.0)	(0.1–37.7, 1.0–7.1)	
Triangular Index *	12.3	15.7	11.7	0.21
	(4.1–33.3, 9.6–17.3)	(6.7–33.3, 10.8–20.1)	(4.1–28.4, 9.5–16.2)	

* value expressed as median with range and also interquartile range. ^a^ Four high risk patients were excluded from the pre-screened consecutive 166 individuals. ^b^ For age, the range is additionally shown. APE—acute pulmonary embolism; BMI—body mass index; HR—heart rate; SBP—systolic blood pressure; LVDD—left ventricle diastolic diameter; LVEF—left ventricle ejection fraction; RVD—right ventricle diameter; NT-proBNP—N-terminal pro-B-type natriuretic peptide; TRPG—tricuspid regurgitation peak gradient; RVSP—right ventricular systolic pressure; IVC—inferior vena cava diameter; AcT—right ventricular outflow Doppler acceleration time; TAPSE—tricuspid annulus plane systolic excursion; SDNN—standard deviation of intervals of all normal beats; SDNN-I—the average of the standard deviations of N-N intervals for each 5 min; SDANN—standard deviation of five-minute mean N-N interval; pNN50—percentage of intervals that are more than 50 ms different from previous interval; RMSSD—root mean square standard deviation; triangular index—integral of the density of the NN interval histogram divided by its height; Bold values show significant results.

**Table 2:** Correlations between time-domain heart rate variability indices, right ventricle overload echocardiographic parameters and also N-terminal pro B type natriuretic peptide concentration in all low and intermediate risk patients with acute pulmonary embolism (*n* = 162) ^a^.

	Mean HR	SDNN (ms)	SDNN-I (ms)	SDANN (ms)	RMSSD (ms)	pNN50 (%)	Triangular Index
TRPG (mmHg)	r = 0.08, *p* = 0.36	r = −0.22, *p* = 0.02	r = −0.14, *p* = 0.11	r = −0.16, *p* = 0.07	r = −0.30, *p* = 0.003	r = −0.80, *p* = 0.41	r = −0.19, *p* = 0.04
RVSP (mmHg)	r = 0.09, *p* = 0.33	r = −0.31, *p* = 0.001	r = −0.21, *p* = 0.03	r = −0.31, *p* = 0.001	r = −0.80, *p* = 0.45	r = −1.10, *p* = 0.29	r = −0.28, *p* = 0.003
TAPSE (mm)	r = −0.11, *p* = 0.11	r = 0.21, *p* = 0.03	r = 0.09, *p* = 0.39	r = 0.27, *p* = 0.006	r = 0.50, *p* = 0.61	r = 0.70, *p* = 0.49	r = 0.22, *p* = 0.49
IVC (mm)	r = 0.06, *p* = 0.11	r = −0.27, *p* = 0.002	r = −0.18, *p* = 0.01	r = −0.18, *p* = 0.04	r = −1.00, *p* = 0.31	r = −0.80, *p* = 0.44	r = −0.29, *p* = 0.44
AcT (ms)	r = −0.10, *p* = 0.24	r = 0.31, *p* = 0.001	r = 0.29, *p* = 0.01	r = 0.38, *p* = 0.001	r = 1.60, *p* = 0.12	r = 2.10, *p* = 0.36	r = 0.30, *p* = 0.04
NT-proBNP (pg/mL)	r = 0.22, *p* = 0.03	r = −0.30, *p* = 0.004	r = −0.24, *p* = 0.02	r = −0.35, *p* = 0.001	r = −0.60, *p* = 0.52	r = −1.10, *p* = 0.28	r = −0.26, *p* = 0.01

^a^ four high risk patients were excluded from the pre-screened consecutive 166 individuals. Abbreviations—see Table 1.

**Table 3:** Logistic regression analysis assessing the influence of right ventricle overload echocardiographic parameters and N-terminal pro B type natriuretic peptide concentration on the standard deviation of intervals of all normal beats (SDNN) corrected for its increase of 10 ms.

	Univariate Analysis		Multivariate Analysis *	
	Odds Ratio (95% CI)	*p*-Value	Odds Ratio (95% CI)	*p*-Value
TRPG (mmHg) ^a^	0.52 (0.11–0.92)	0.013	-	-
RVSP (mmHg) ^a^	0.39 (0.26–0.93)	0.001	-	-
TAPSE (mm) ^b^	2.50 (1.18–3.10)	0.027	-	-
IVC (mm) ^b^	0.69 (0.39–0.83)	0.002	0.91 (0.21–0.92)	0.014
AcT (ms) ^c^	1.38 (1.33–5.98)	0.002	-	-
NT-proBNP (pg/mL) ^d^	0.23 (0.18–0.31)	0.002	0.83 (0.04–0.91)	0.041

Abbreviations—see Table 1. * results of multivariate analysis shown only for statistically significant parameters. ^a^ Odds ratio and 95% CI corrected for an increase of 10 mmHg. ^b^ Odds ratio and 95% CI corrected for an increase of 1 mm. ^c^ Odds ratio and 95% CI corrected for an increase of 10 ms. ^d^ Odds ratio and 95% CI corrected for an increase of 100 pg/mL.

## Data Availability

No publicly archived datasets were analyzed or generated during the study.

## References

[1] Konstantinides S.V., Meyer G., Becattini C., Bueno H., Geersing G.J., Harjola V.-P., Huisman M.V., Humbert M., Jennings C.S., Jiménez D. (2020). 2019 ESC Guidelines for the diagnosis and management of acute pulmonary embolism developed in collaboration with the European Respiratory Society (ERS). Eur. Heart J..

[2] Huikuri H.V., Stein P.K. (2013). Heart rate variability in risk stratification of cardiac patients. Prog. Cardiovasc. Dis..

[3] Lisicka M., Radochońska J., Bienias P. (2019). Arrhythmias and autonomic nervous system dysfunction in acute and chronic diseases with right ventricle involvement. Folia Cardiologica.

[4] (1996). Heart Rate Variability: Standards of Measurement, Physiological Interpretation, and Clinical Use. Task Force of the European Society of Cardiology the North American Society of Pacing Electrophysiology. Circulation.

[5] Lang R.M., Badano L.P., Mor-Avi V., Afilalo J., Armstrong A., Ernande L., Flachskampf F.A., Foster E., Goldstein S.A., Kuznetsova T. (2015). Recommendations for cardiac chamber quantification by echocardiography in adults: An update from the American Society of Echocardiography and the European Association of Cardiovascular Imaging. Eur. Heart J. Cardiovasc. Imaging.

[6] Kurnicka K., Lichodziejewska B., Goliszek S., Dzikowska-Diduch O., Zdończyk O., Kozłowska M., Kostrubiec M., Ciurzyński M., Palczewski P., Grudzka K. (2016). Echocardiographic Pattern of Acute Pulmonary Embolism: Analysis of 511 Consecutive Patients. J. Am. Soc. Echocardiogr..

[7] Hayano J., Yuda E. (2019). Pitfalls of assessment of autonomic function by heart rate variability. J. Physiol. Anthropol..

[8] Shaffer F., Ginsberg J.P. (2017). An Overview of Heart Rate Variability Metrics and Norms. Front. Public Health.

[9] Bienias P., Kostrubiec M., Rymarczyk Z., Korczak D., Ciurzyński M., Kurzyna M., Torbicki A., Fijałkowska A., Pruszczyk P. (2015). Severity of arterial and chronic thromboembolic pulmonary hypertension is associated with impairment of heart rate turbulence. Ann. Noninvasive Electrocardiol..

[10] Bienias P., Ciurzynski M., Kostrubiec M., Rymarczyk Z., Kurzyna M., Korczak D., Roik M., Torbicki A., Fijalkowska A., Pruszczyk P. (2015). Functional class and type of pulmonary hypertension determinate severity of cardiac autonomic dysfunction assessed by heart rate variability and turbulence. Acta Cardiol..

[11] Witte C., Meyer Zur Heide Genannt Meyer-Arend J.U., Andrié R., Schrickel J.W., Hammerstingl C., Schwab J.O., Nickenig G., Skowasch D., Pizarro C. (2016). Heart Rate Variability and Arrhythmic Burden in Pulmonary Hypertension. Adv. Exp. Med. Biol..

[12] Peters E.L., Bogaard H.J., Vonk Noordegraaf A., de Man F.S. (2021). Neurohormonal modulation in pulmonary arterial hypertension. Eur. Respir. J..

[13] Stratmann G., Gregory G.A. (2003). Neurogenic and humoral vasoconstriction in acute pulmonary thromboembolism. Anesth. Analg..

[14] Paula-Ribeiro M., Ribeiro I.C., Aranda L.C., Silva T.M., Costa C.M., Ramos R.P., Ota-Arakaki J.S., Cravo S.L., Nery L.E., Stickland M.K. (2019). Carotid chemoreflex activity restrains post-exercise cardiac autonomic control in healthy humans and in patients with pulmonary arterial hypertension. J. Physiol..

[15] Okólska M., Łach J., Matusik P.T., Pająk J., Mroczek T., Podolec P., Tomkiewicz-Pająk L. (2021). Heart Rate Variability and Its Associations with Organ Complications in Adults after Fontan Operation. J. Clin. Med..

[16] Folsom A.R., Lutsey P.L., Pope Z.C., Fashanu O.E., Misialek J.R., Cushman M., Michos E.D. (2019). Atherosclerosis Risk in Communities (ARIC) Study Investigators. Resting heart rate and incidence of venous thromboembolism. Res. Pract. Thromb. Haemost..

[17] Awotoye J., Fashanu O.E., Lutsey P.L., Zhao D., O’Neal W.T., Michos E.D. (2020). Resting heart rate and incident venous thromboembolism: The Multi-Ethnic Study of Atherosclerosis. Open Heart.

[18] Khan A.A., Lip G.Y.H., Shantsila A. (2019). Heart rate variability in atrial fibrillation: The balance between sympathetic and parasympathetic nervous system. Eur. J. Clin. Invest..

[19] Ng A.C., Adikari D., Yuan D., Lau J.K., Yong A.S., Chow V., Kritharides L. (2016). The Prevalence and Incidence of Atrial Fibrillation in Patients with Acute Pulmonary Embolism. PLoS ONE.

[20] Krajewska A., Ptaszynska-Kopczynska K., Kiluk I., Kosacka U., Milewski R., Krajewski J., Musial W.J., Sobkowicz B. (2017). Paroxysmal Atrial Fibrillation in the Course of Acute Pulmonary Embolism: Clinical Significance and Impact on Prognosis. Biomed. Res. Int..

[21] Majos E., Dąbrowski R., Szwed H. (2013). The right ventricle in patients with chronic heart failure and atrial fibrillation. Cardiol. J..

[22] Radochońska J., Lisicka M., Bienias P. (2019). The purpose of electrocardiography in acute and chronic diseases with right ventricular involvement. Folia Cardiol..

[23] Keller K., Beule J., Balzer J.O., Dippold W. (2016). Syncope and collapse in acute pulmonary embolism. Am. J. Emerg. Med..

[24] Liesching T., O’Brien A. (2002). Significance of a syncopal event. Pulmonary embolism. Postgrad. Med..

